# Efficacy and Safety of Tislelizumab as an Immune Checkpoint Inhibitor for the Treatment of Advanced Hepatocellular Carcinoma: A Systematic Review and Meta-Analysis

**DOI:** 10.7759/cureus.87664

**Published:** 2025-07-10

**Authors:** Chao Yuan Li Cai, Abigail Rai, Hariharasudhan Balaji, Arvin Raju, Shruthi Murugan, Shyam Nikethen Girivasan, Samuel Rai

**Affiliations:** 1 General Medicine, Hywel Dda Health Board, Carmarthen, GBR; 2 Acute Medical Unit, Impur Christian Hospital, Impur, IND; 3 Emergency Medicine, Southern Health and Social Care Trust, Portadown, GBR; 4 Internal Medicine, Arthur Hospital, Nagapattinam, IND; 5 Bariatric and Minimal Access Surgery, Manipal Hospital Millers Road, Bengaluru, IND; 6 Pharmacy, JSS University College of Pharmacy, Ooty, IND; 7 Medicine, ACS Medical College and Hospital, Chennai, IND

**Keywords:** hepatocellular carcinoma (hcc), immune checkpoint inhibitors, objective response rate, progression-free survival (pfs), systematic review and meta-analysis

## Abstract

Hepatocellular carcinoma (HCC) remains a leading global cause of cancer-related mortality, necessitating effective systemic therapies. Tislelizumab, a novel anti-PD-1 monoclonal antibody, has shown promise in recent trials. However, a pooled assessment of its clinical efficacy and safety in HCC treatment has been lacking. To systematically evaluate and quantify the efficacy and safety of tislelizumab in HCC, focusing on key outcomes including overall survival (OS), progression-free survival (PFS), objective response rate (ORR), and treatment-related adverse events (TRAEs), a systematic search of PubMed, Scopus, Cochrane Library, ScienceDirect, the American Society of Clinical Oncology (ASCO), and the European Society of Medical Oncology (ESMO) databases was conducted for clinical trials from January 2010 to October 2024. Seven studies involving 1,178 patients were included in the analysis. Statistical analyses were performed using the Comprehensive Meta-Analysis software (version 3.7). Fixed-effect and random-effects models were used based on heterogeneity (I² statistic), and publication bias was assessed using Egger's and Begg's tests, alongside Duval and Tweedie's trim-and-fill method. The pooled ORR was estimated at 22.3% (logit = -1.242; SE = 0.0897), but after correcting for publication bias (Egger's p = 0.027), the adjusted ORR decreased to 17.2%. The pooled OS was 13.66 months (95% CI: 11.97-15.35) with no heterogeneity (I² = 0%), affirming the robustness of the survival benefit. PFS was 4.58 months under a fixed-effect model but increased to 5.86 months with the random-effects model due to high heterogeneity (I² = 93.15%). The incidence of Grade ≥ 3 TRAEs was 28.9% (logit = -0.448), indicating a manageable safety profile, with no significant publication bias (Egger's p = 0.251). Tislelizumab demonstrates favorable clinical activity in HCC, offering meaningful improvements in survival and disease control with acceptable toxicity. While ORR results were influenced by potential publication bias, survival and safety outcomes were consistent and reliable. These findings support tislelizumab as a viable immunotherapeutic option for advanced HCC, warranting further exploration in larger, randomized trials and combination therapy regimens to optimize patient outcomes.

## Introduction and background

Hepatocellular carcinoma (HCC) is the most common primary liver tumor and the fifth most common cancer worldwide, constituting over 90% of liver cancers and predominantly occurring in patients with cirrhosis, carrying an annual HCC incidence of 2-4%, and having a dismal five-year survival rate of 18% [1]. Key etiology includes chronic viral hepatitis (hepatitis B and C), alcoholic liver disease, and non-alcoholic steatohepatitis/non-alcoholic fatty liver disease, emphasizing the critical need for early detection and management [2]. Cirrhosis plays a pivotal role in viral carcinogenesis for HCC, driven by hepatitis B virus genome integration into telomerase reverse transcriptase promoter sites and genetic mutations (e.g., *TP53*, *CTNNB1*, *ARID1A*), while chronic hepatitis C virus infection contributes through inflammation, fibrosis, and viral proteins, predominantly in cirrhotic or advanced fibrosis cases [1,3].

The advent of immunotherapies targeting the programmed death receptor-1 (PD-1) pathway has transformed the treatment landscape of various solid tumors [4]. Tislelizumab, a humanized anti-PD-1 monoclonal antibody, is designed to minimize binding to Fcγ receptors on macrophages, exhibiting high affinity and prolonged receptor engagement. Its unique binding properties enhance its inhibitory effects on PD-1/PD-L1 signaling, potentially improving anti-tumor immune responses [5-7].

Tislelizumab has demonstrated a favorable safety profile and manageable treatment-related adverse events (TRAEs) across multiple clinical trials in non-HCC solid tumors, showing effectiveness in treating advanced lung cancer [8], non-small cell lung cancer (NSCLC) subtypes [9], and EGFR + TP53 co-variant lung adenocarcinoma [10], both as monotherapy and in combination with chemotherapy, supporting the drug's general immunotherapeutic potential. Its efficacy is highlighted by improved health-related quality of life, superior clinical outcomes, and cost-effectiveness compared to standard chemotherapy, making it a promising treatment option despite limitations in the generalizability and heterogeneity of current trial data [11]. Tislelizumab received its first regulatory approval from China's National Medical Products Administration (NMPA) in December 2019 for relapsed or refractory classical Hodgkin's lymphoma [7], followed by approvals for metastatic urothelial carcinoma with high PD-L1 expression in 2020 [12] and for NSCLC (with chemotherapy) and hepatocellular carcinoma (as monotherapy) in 2021 [13]. Additionally, applications for second-line NSCLC therapy and MSI-H/dMMR solid tumors have been accepted, highlighting tislelizumab's expanding indications and promising anti-tumor efficacy [5]. Several clinical trials [9,14-16] have shown promising results in the efficacy and safety of tislelizumab for the treatment of HCC; however, there is no meta-analysis analyzing the pooled outcomes to measure its efficacy in this particular disease condition.

Aims and objectives

This study aims to systematically evaluate and assess the efficacy and safety of tislelizumab as an immune checkpoint inhibitor in the treatment of HCC. Thus, the primary objective of this study was to systematically evaluate and quantify the efficacy and safety of tislelizumab, a PD-1 inhibitor, in the treatment of HCC by synthesizing data from clinical trials. Given the rising interest in immune checkpoint inhibitors for HCC and the emergence of tislelizumab as a promising agent, this meta-analysis aimed to generate pooled estimates for key clinical outcomes, including objective response rate (ORR), overall survival (OS), progression-free survival (PFS), and TRAEs. This study aims to provide a comprehensive understanding of tislelizumab's therapeutic potential, highlighting its effectiveness in improving clinical outcomes and addressing safety concerns in patients with HCC.

## Review

Methodology

This study was conducted following the Preferred Reporting Items for Systematic Reviews and Meta-Analyses (PRISMA) guidelines [17] for conducting this systematic review and meta-analysis.

Search Strategy

Two reviewers comprehensively searched for eligible studies from MEDLINE (PubMed), Central (Cochrane Library), Scopus, ScienceDirect, the American Society of Clinical Oncology (ASCO), and the European Society of Medical Oncology (ESMO) databases from January 2010 to October 2024, focusing on randomized controlled trials (RCTs) and clinical trials identifying the efficacy and safety assessment of tislelizumab. Table 1 provides a detailed description of the keywords with Boolean operators used for each database. No language restriction was applied. The reference lists of all eligible trials were also searched to identify other studies. Duplicate studies were eliminated using EndNote 20.2.1 (Clarivate Analytics, Philadelphia, PA). The electronic databases PubMed and Cochrane Library were selected for their extensive coverage of clinical and systematic reviews, providing relevant peer-reviewed studies on imaging techniques.

**Table 1:** Search strategy employed

Database	Search Terms
PubMed	(("Tislelizumab"[Title/Abstract] OR "BGB-A317"[Title/Abstract] OR "anti-PD-1 antibody"[Title/Abstract] OR "anti-PD-1 therapy"[Title/Abstract]) AND ("hepatocellular carcinoma"[Title/Abstract] OR "liver cancer"[Title/Abstract] OR "HCC"[Title/Abstract])) AND (clinicaltrial[Filter] OR randomizedcontrolledtrial[Filter])
Cochrane Library	(Tislelizumab) AND (hepatocellular carcinoma OR liver cancer OR HCC)
Scopus	( TITLE-ABS-KEY ( tislelizumab ) AND TITLE-ABS-KEY ( "hepatocellular carcinoma" OR HCC ) AND TITLE-ABS-KEY ( "treatment-related adverse events" OR TRAEs ) )
ScienceDirect	( tislelizumab ) AND ( "hepatocellular carcinoma" OR HCC ) AND ( "treatment-related adverse events" OR TRAEs )
American Society of Clinical Oncology (ASCO)	"tislelizumab" AND "Hepatocellular carcinoma"
European Society of Medical Oncology (ESMO)	"tislelizumab"

Study Selection

The study selection criteria for this meta-analysis followed the SPIDER framework to accommodate both single-arm and comparative studies assessing tislelizumab for HCC. The sample included adult patients (≥18 years) with a confirmed diagnosis of HCC at any stage (early, intermediate, or advanced). The phenomenon of interest focused on evaluating the clinical efficacy and safety of tislelizumab, including outcomes such as OS, PFS, ORR, and TRAEs. Studies with various designs, such as RCTs, prospective or retrospective cohort studies, and single-arm trials, were included to capture a broad spectrum of clinical evidence. The evaluation centered on studies reporting at least one predefined outcome, with data presented as event rates, hazard ratios (HR), means and standard deviations (SD), or proportions. Both comparative studies (e.g., tislelizumab vs. placebo or standard therapies) and single-arm studies providing sufficient outcome data were eligible for inclusion. The framework emphasized research types focused on quantitative studies that report survival outcomes or adverse event proportions. Reviews, case reports, editorials, conference abstracts, and studies without relevant outcome data were excluded. The SPIDER framework facilitated comprehensive and flexible study selection, allowing for the inclusion of diverse study designs and data to evaluate the efficacy and safety of tislelizumab in HCC treatment.

Data Collection and Quality Evaluation

Two reviewers independently assessed the papers on EndNote 20.2.1 based on their titles and abstracts, in accordance with the qualifying requirements. EndNote 20.2.1 was chosen for title and abstract screening due to its efficient reference management capabilities and automatic duplicate detection, which streamlined the initial study selection process. The full texts of the remaining papers were separately reviewed, and disagreements were resolved by a third, blinded reviewer who independently made a final decision on disputed studies, ensuring an unbiased resolution process.

The following information was retrieved from the studies using a piloted Microsoft Excel (Microsoft Corporation, Redmond, Washington) sheet: the first author's surname with year of publication, country of study, study design, diagnosis of the cohort, number of participants (n), mean age with female count and percentage, clinical interventions used, and measured outcomes. Microsoft Excel was used for data extraction due to its flexibility in organizing multiple categories and filtering data, which allowed for detailed categorization across study characteristics and outcomes. The data extraction sheet in Excel was pre-tested with a sample of studies to ensure consistency in data entry and category definitions. The sheet included key categories such as author, publication year, study design, sample size, mean age, diagnostic outcomes, and quality assessment scores. Additionally, specific columns were dedicated to quality notes, enabling systematic documentation of methodological rigor and potential sources of bias. The study quality was scaled. The quality of evidence for each included study was evaluated using the GRADE (Grading of Recommendations Assessment, Development, and Evaluation) approach [18]. This framework assesses evidence based on factors including study design, risk of bias, inconsistency, indirectness, imprecision, and potential publication bias. Each study was assigned a certainty rating: high, moderate, low, or very low, depending on its methodological rigor and relevance to the research question.

For the included RCTs, the Cochrane Risk of Bias 2 (Rob2) tool [19] was used for assessing the risk of bias whereby studies were assessed based on the following five domains: (i) the randomization process; (ii) deviations from intended interventions; (iii) missing outcome data; (iv) measurement of the outcome; and (v) selection of the reported result. For the included observational or cohort studies, the Cochrane Risk of Bias in Non-Randomized Studies - of Interventions (ROBINS-I) tool [20] was used, where the studies were assessed based on the seven domains of confounding, selection of participants, classification of interventions, deviations from intended interventions, missing data, measurement of outcomes, and selection of reported results. The results were represented through traffic light plots and summary bar charts using the Cochrane Risk-of-bias VISualization (Robvis) tool [21]. In the traffic light plot, green, yellow, and red indicators represented low, moderate, and high risk of bias, respectively, across domains. Expected findings included a low to moderate risk of bias in most domains, with occasional high bias in participant selection. Bar charts summarized these findings, highlighting areas where specific biases were prevalent across studies. To mitigate potential biases, we employed rigorous inclusion criteria, ensured consistency in data extraction, and performed sensitivity analyses wherever feasible.

Statistical Analysis

The statistical analysis was performed using Comprehensive Meta-Analysis (CMA) software, version 3.7, which facilitated the estimation of pooled effects under both fixed-effects and random-effects models. The fixed-effect model was initially applied to provide a weighted average effect, assuming homogeneity across studies. In contrast, the random-effects model was subsequently employed when heterogeneity was detected, allowing for between-study variance and ensuring a more conservative estimate. Specifically, fixed and random models were used for ORR, OS, PFS, and TRAEs, depending on heterogeneity metrics. Heterogeneity was assessed using Cochran's Q statistic and I² index, where I² values above 50% indicated moderate to high heterogeneity. For ORR (I² = 89.93%), PFS (I² = 93.15%), and TRAEs (I² = 97.2%), high heterogeneity justified the use of the random-effects model. In contrast, OS showed no significant heterogeneity (I² = 0%), validating the fixed-effect model. To evaluate publication bias, the analysis incorporated several tests: Begg and Mazumdar's rank correlation (Kendall's tau-b), Egger's regression intercept test, and Duval and Tweedie's trim-and-fill method. ORR exhibited evidence of publication bias (Egger's p = 0.027), while OS, PFS, and TRAEs did not. Fail-safe N and Orwin's fail-safe N further assessed the robustness of pooled estimates by estimating the number of missing null studies required to nullify observed effects. Collectively, this methodological framework ensured a rigorous and statistically sound evaluation of tislelizumab's clinical outcomes across diverse studies.

Results

PRISMA Compliance

**Figure 1:**
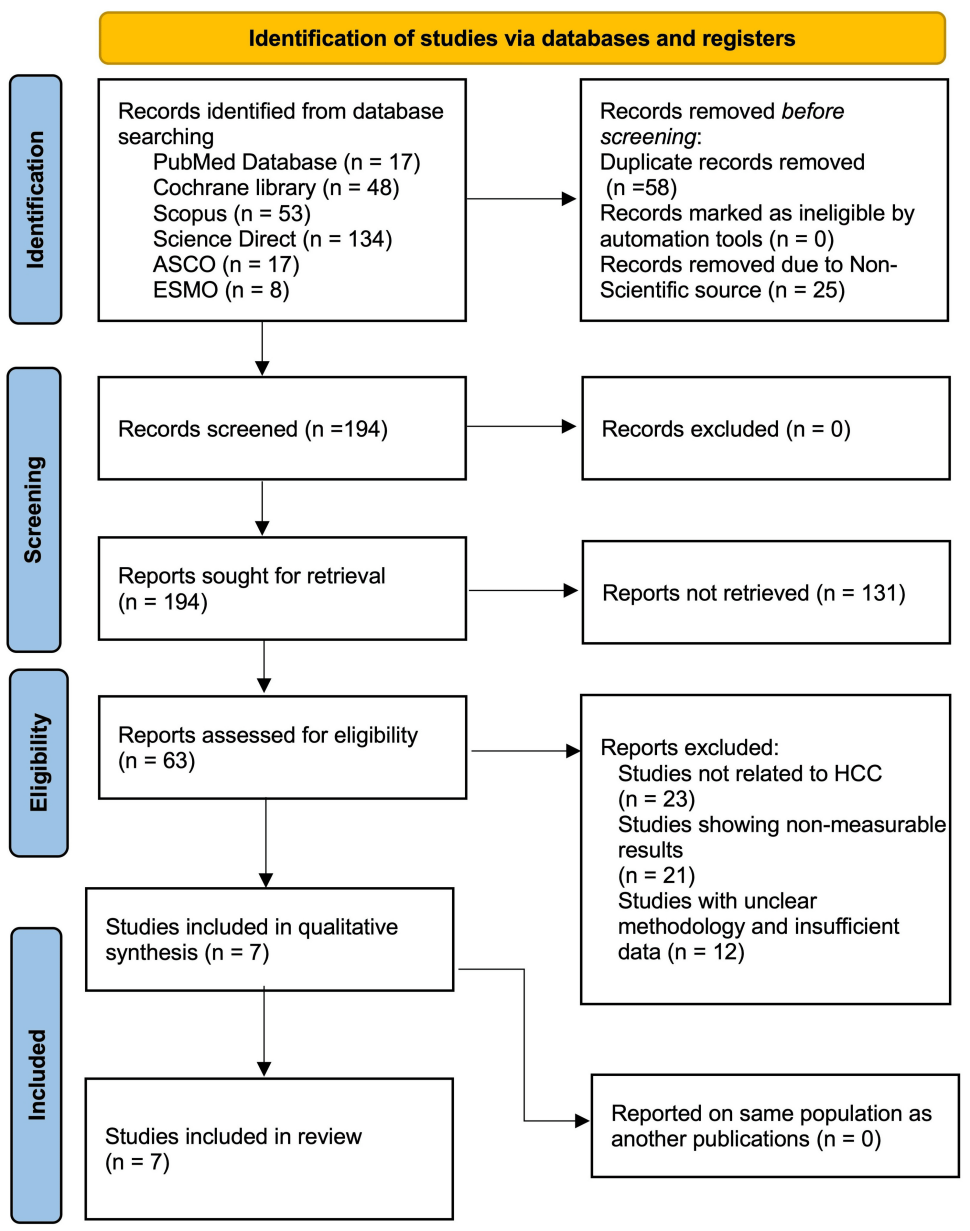
PRISMA flowchart PRISMA: Preferred Reporting Items for Systematic Reviews and Meta-Analyses

The PRISMA flowchart in Figure 1 visually represents the systematic process of literature identification, screening, eligibility assessment, and inclusion for this meta-analysis on tislelizumab in HCC. A total of 277 records were identified through six major databases, including PubMed (n=17), Cochrane Library (n=48), Scopus (n=53), ScienceDirect (n=134), ASCO (n=17), and ESMO (n=8). Prior to screening, 83 records were removed, comprising 58 duplicates and 25 non-scientific sources, including news reports and non-peer-reviewed content. After these exclusions, 194 records were screened, all of which were considered for full-text retrieval. A total of 131 full-text reports could not be retrieved, leaving 63 studies to be assessed for eligibility. Of these, 56 studies were excluded, as 23 were not related to HCC, 21 presented non-measurable results, and 12 lacked sufficient methodological clarity or data. This process resulted in the inclusion of seven studies in both the qualitative synthesis and final review. No additional studies were excluded for reporting on overlapping populations.

Study Characteristics

The included studies in this systematic review represent a diverse range of clinical designs and populations evaluating tislelizumab in the treatment of HCC, as shown in Table 2. A total of seven studies were analyzed, encompassing RCTs, phase I/II single-arm trials, and multicenter studies. The sample sizes ranged from 20 to 674 patients, with varying age distributions (mean age between 52.5 and 62 years) and a predominantly male population, reflecting the epidemiology of HCC. Most studies have focused on advanced or unresectable HCC, with some investigating tislelizumab as monotherapy, while others have assessed it in combination with agents such as radiotherapy, lenvatinib, or bevacizumab biosimilars. Geographically, the majority of studies were conducted in China, while two were global or multi-regional in scope.

**Table 2:** Study characteristic table HCC: hepatocellular carcinoma; ORR: objective response rate; CR: complete response; PR: Partial response; SD: stable disease; PD: progressive disease; DCR: disease control rate; OS: overall survival; PFS: progression-free survival; AE: adverse events; pRR: pathological response rate; pCR: pathological complete response; MPR: major pathological response; RFS: recurrence-free survival; SBRT: stereotactic body radiotherapy; IMRT: intensity-modulated radiotherapy; uHCC: unresectable hepatocellular carcinoma

Study	Country	Design	Diagnosis	Patient Count	Mean Age (Years)	Female % (n)	Intervention	Outcomes Reported
Ren et al. 2022 [22]	Eight countries in Europe and Asia	Non-randomized, multicenter, open-label phase 2	Advanced HCC	249	62	12.9% (32)	Tislelizumab 200 mg i.v. every 3 weeks, on day 1 of each 21-day cycle	ORR, duration of response, DCR, OS, AE
Qin et al. 2023 [23]	Global, multiregional	Open-label, phase 3, randomized clinical trial	HCC	674	61	15.4% (104)	Tislelizumab, 200 mg intravenously every 3 weeks vs. sorafenib tosylate, 400 mg orally twice daily	OS, ORR, PFS, duration of response, AE
Ren et al. 2023 [24]	China	Phase 2, randomized open-label study	HCC	94	58.5	NA	Ociperlimab (OCI) + tislelizumab (TIS) + BAT1706 (bevacizumab biosimilar) versus TIS + BAT1706	OR, CR, PR, SD, PD, ORR, PFS, duration of response, AE
Li Z et al. 2023 [25]	China	Single-center phase 1b	Early-stage HCC	20	58.5	NA	SBRT plus tislelizumab	ORR, pRR, pCR, AE
Li J et al. 2024 [26]	China	Single-center, single-arm, phase II clinical trial	Advanced HCC	47	53	10.6% (5)	Sitravatinib ± tislelizumab	OR, DCR, OS, PFS, AE
Pan et al. 2024 [27]	China	Single-arm, phase 2 trial	Advanced HCC with macrovascular invasion	30	NA	13.3% (4)	Tislelizumab plus IMRT	ORR, OS, pCR/MPR, RFS, AE
Xu et al. 2024 [28]	China	Prospective, multicenter, open-label, single-arm, phase 2 study	Systemic treatment-naïve patients with uHCC	64	52.5	17.2% (11)	Tislelizumab plus lenvatinib	ORR, CR, PR, DCR, AE

GRADE Assessment

The study by Qin et al. (2023) [23] was a phase III RCT comparing tislelizumab to sorafenib. It was the only study rated as high-quality evidence according to the GRADE framework due to its robust design and sample size. In contrast, early-phase single-arm studies, including Ren et al. (2022) [22], Li et al. (2023) [25], and Pan et al. (2024) [27], were rated as very low to low quality due to small sample sizes, lack of control groups, and indirect applicability to broader HCC populations. Intermediate-quality evidence was derived from Ren et al. (2023) [24] and Xu et al. (2024) [28], which, despite their moderate designs, had limitations related to imprecision and indirectness. The full GRADE assessment of each study, outlining factors such as risk of bias and consistency, is detailed as a supplementary table in the Appendices (Table 3). This characterization facilitates the interpretation of pooled outcomes and highlights the need for caution when generalizing findings from lower-certainty evidence.

Risk of Bias Assessment

**Figure 2:**
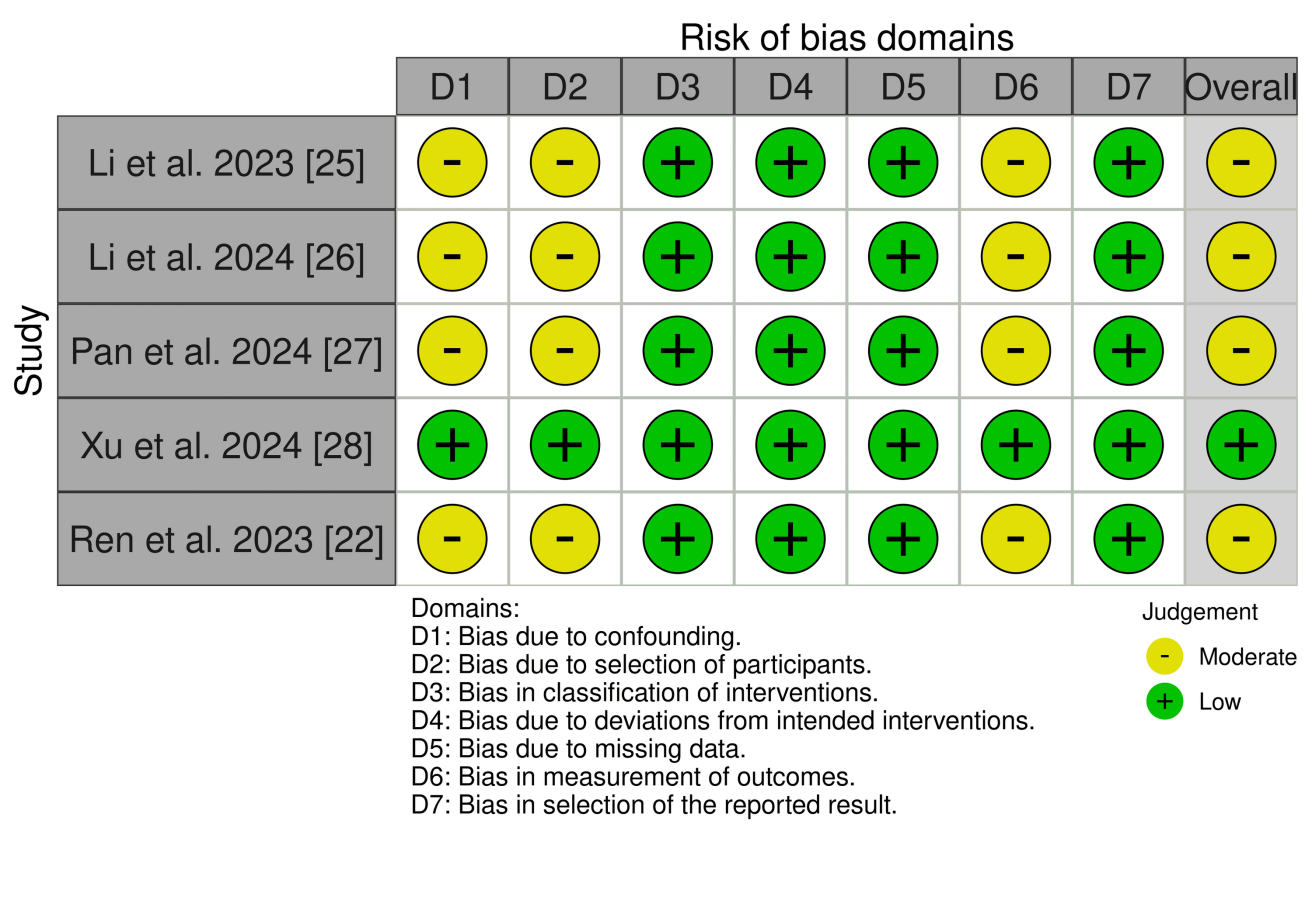
Risk of bias of the included non-randomized studies

The risk of bias across seven domains for the five non-randomized cohort studies indicates an overall moderate risk, as depicted in Figure 2. Moderate risks were noted in confounding (D1) for Li et al. [25] and Ren et al. [22], primarily due to inadequate adjustment for critical variables, such as tumor stage, prior treatments, or patient comorbidities, which could potentially skew the outcomes. Similarly, the selection of participants (D2) posed a moderate risk in Li et al. [25], Pan et al. [27], and Ren et al. [22], likely due to non-randomized designs and imbalanced baseline characteristics, which limited cohort comparability. All studies exhibited a moderate risk in outcome measurement (D6) due to the subjective nature of certain outcomes, such as tumor response or adverse event severity, which may not have been assessed in a blinded manner, potentially leading to observer bias. Additionally, selective reporting (D7) was evident across all studies, raising concerns about transparency as non-significant or unfavorable results may have been excluded. Conversely, the studies demonstrated low risk in intervention classification (D3), deviations from interventions (D4), and missing data (D5), reflecting sound methodological execution in these domains. The overall moderate risk of bias underscores the need for future studies to implement rigorous design measures, such as randomization or propensity score matching, blinded outcome assessments, and pre-registration of study protocols, to ensure robust and reliable evaluation of tislelizumab's efficacy and safety in hepatocellular carcinoma.

**Figure 3:**
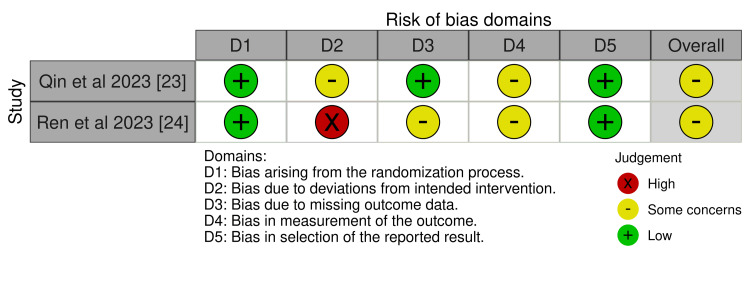
Risk of bias of the included randomized controlled studies

The risk of bias assessment in Figure 3 for the studies "Qin et al. (2023) [23]" and "Ren et al. (2023) [24]" highlights several methodological strengths and limitations. Qin et al. (2023) demonstrated a low risk of bias in domains related to the randomization process (D1), outcome measurement (D4), and selection of reported results (D5). However, there were "some concerns" regarding deviations from intended interventions (D2), potentially indicating minor protocol inconsistencies, and missing outcome data (D3), which may suggest incomplete follow-ups or insufficient data handling. The overall risk of bias for this study was deemed "some concerns." Ren et al. (2023) [24], similarly, showed a low risk in D1 and D5, confirming proper randomization and comprehensive reporting. However, it presented a high risk of bias in D2 due to significant deviations from the intended interventions, which could potentially impact the study's validity. Additionally, "some concerns" were noted for D3 and D4, reflecting issues such as incomplete data and subjective measurements. Overall, while both studies provide valuable insights into tislelizumab's efficacy, concerns about deviations from interventions, missing data, and subjective assessments underscore the need for cautious interpretation of their findings.

Objective Response Rate

**Figure 4:**
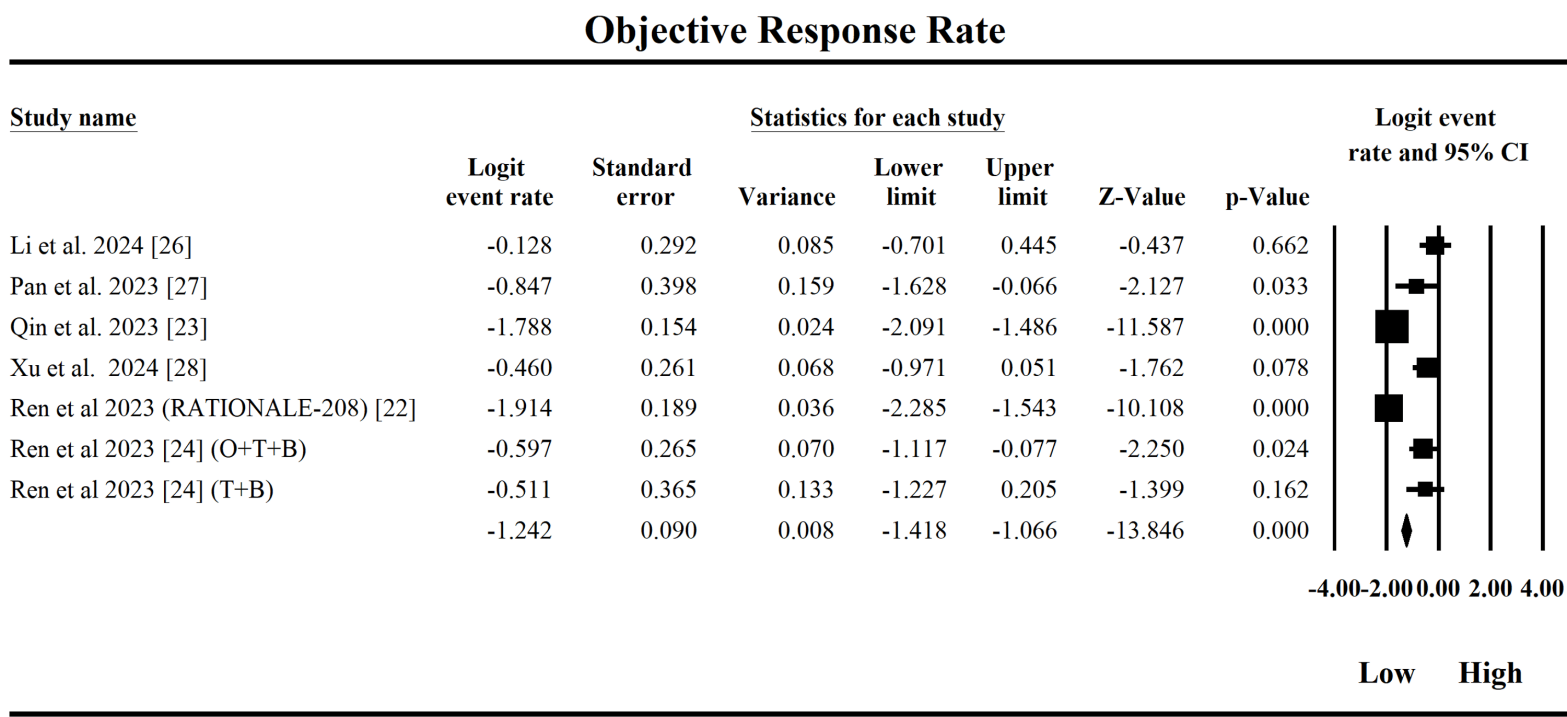
Forest plot depicting objective response rate (ORR) O+T+B: ociperlimab (O) + tislelizumab (T) + BAT1706 (B); T+B: tislelizumab (TIS) + BAT1706 (B); CI: confidence interval

The forest plot in Figure 4 evaluates the ORR of tislelizumab in treating HCC by synthesizing data from six studies. The fixed-effect model yielded a pooled logit effect size of -1.242 (standard error = 0.0897), corresponding to an estimated ORR of approximately 22.3%, indicating that the response rate to tislelizumab across these studies remains modest but clinically relevant. A logit value of -1.242 translates to a probability of response of 0.223, meaning that approximately 22.3% of patients with HCC responded to the drug. Key contributors to this overall effect include studies by Qin et al. (2023) [23] and Ren et al. (2023) [22], both showing more pronounced negative logits (less favorable response), with logit values of -1.79 and -1.91, respectively, and associated p-values of 0, indicating statistically significant effects. Conversely, Li et al. (2024) [26] and Xu et al. (2024) [28] reported smaller logit values with wider confidence intervals, indicating a lower impact and variability. Importantly, heterogeneity across studies is high, as indicated by an I² of 89.93% and a significant Q-value (59.56, p < 0.0001), suggesting considerable variability not due to chance. The random-effects model, adjusting for this heterogeneity, yielded a slightly less negative pooled logit of -0.920, translating to a higher ORR (~28.5%). Clinically, the findings suggest tislelizumab offers a modest but consistent therapeutic benefit for HCC patients, albeit with variability influenced by study design, population, or tumor characteristics. This supports its role as a viable immunotherapy option while underscoring the need for personalized treatment strategies and further stratified research to optimize response.

Overall Survival

**Figure 5:**
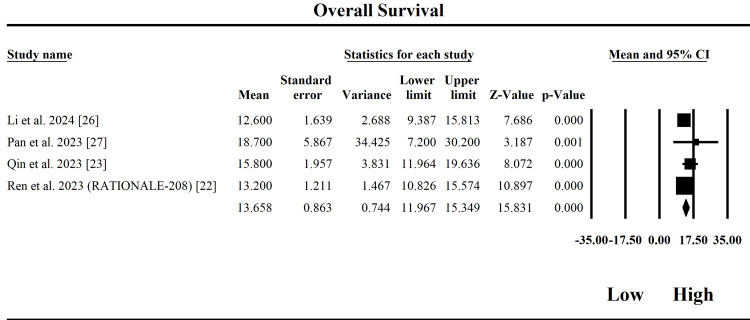
Forest plot of the pooled analysis on overall survival (OS) CI: confidence interval

The forest plot in Figure 5 evaluates the OS outcome of tislelizumab in patients with HCC, based on four included studies. The fixed-effect and random-effects models yielded an identical pooled effect size of 13.66 months (SE = 0.863), with a 95% confidence interval ranging from 11.97 to 15.35 months. The point estimate of 13.66 months represents the average overall survival duration following tislelizumab treatment, indicating that the drug provides a meaningful survival benefit in advanced HCC populations. The analysis revealed no significant heterogeneity across studies (Q = 2.50, df = 3, p = 0.476), with an I² of 0%, suggesting that the variation in outcomes is likely due to chance rather than study-level differences. Additionally, Tau² = 0, confirming the robustness of the findings across studies. The Z-value of 15.83 (p < 0.001) supports that the observed pooled effect is highly statistically significant. Among the individual studies, Qin et al. (2023) [23] and Ren et al. (2023) [22] reported OS durations of 15.8 and 13.2 months, respectively, both with tight confidence intervals and highly significant p-values. Pan et al. (2023) [27] reported the highest OS estimate (18.7 months), but with a large standard error, which reduced its weight in the pooled analysis. Clinically, these results suggest that tislelizumab can extend survival in HCC patients by over a year, offering a compelling treatment option for a population with a historically poor prognosis. Given the consistency across studies and the lack of heterogeneity, these findings are reliable and generalizable, supporting broader integration of tislelizumab into HCC treatment protocols.

Progression-Free Survival

**Figure 6:**
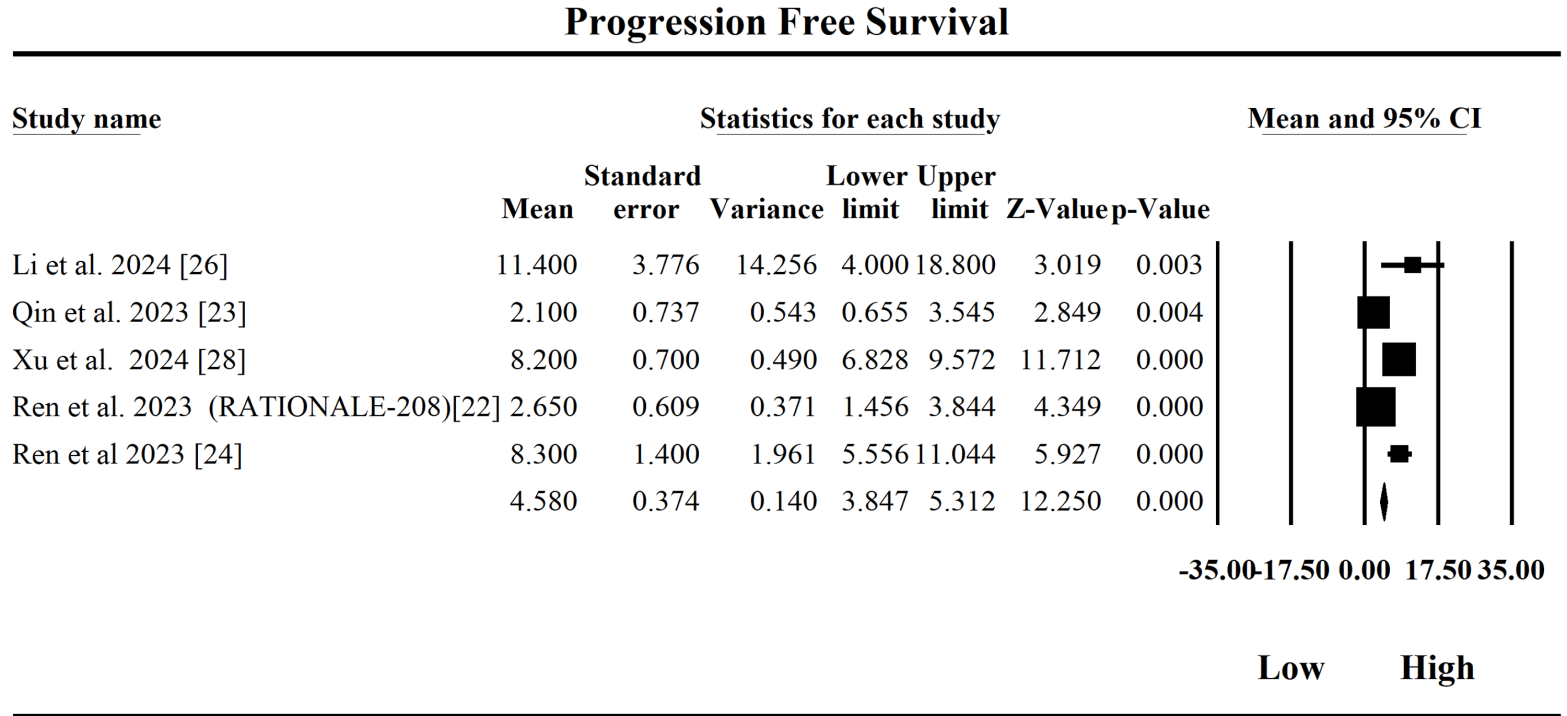
Forest plot of the pooled analysis on progression-free survival (PFS) CI: confidence interval

The forest plot in Figure 6 represents the pooled meta-analysis of the PFS outcomes of tislelizumab in the treatment of HCC across five studies. The fixed-effect model yields a pooled PFS estimate of 4.58 months (95% CI: 3.85-5.31; SE = 0.37), indicating that patients treated with tislelizumab typically experience about 4.6 months without disease progression. This value represents the weighted average duration patients benefit from the treatment before tumor growth or spread occurs. However, the analysis shows substantial heterogeneity (Q = 58.41, df = 4, p < 0.0001), with a very high I² of 93.15%, reflecting significant variability among study outcomes. This heterogeneity is further supported by a high Tau² of 10.78, suggesting the true effects vary considerably across populations and settings. The random-effects model, which accounts for this variability, presents a higher pooled PFS of 5.86 months (95% CI: 2.70-9.02), suggesting some studies observed considerably better outcomes. Among individual studies, Li et al. (2024) [26] and Ren et al. (2023) [24] reported longer PFS durations (11.4 and 8.3 months, respectively), while Qin et al. (2023) [23] and Ren et al. (2023) [22] showed shorter PFS (2.1 and 2.65 months), which significantly influenced overall heterogeneity. Clinically, these findings suggest tislelizumab offers a moderate PFS benefit in HCC, with outcomes varying across patient subgroups. The presence of heterogeneity implies that factors such as tumor burden, prior treatments, and patient immune profiles may significantly affect therapeutic response. These results support its continued use but highlight the importance of patient selection and personalized treatment planning in maximizing clinical benefit.

Treatment-Related Adverse Events

**Figure 7:**
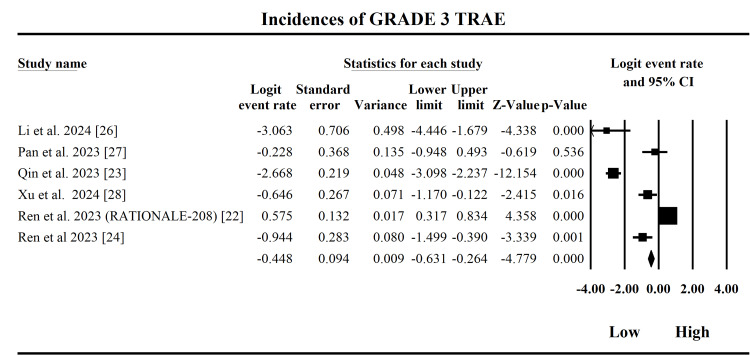
Forest plot on the treatment-related adverse events (TRAEs) pooled data GRADE: Grading of Recommendations Assessment, Development, and Evaluation; CI: confidence interval

The forest plot in Figure 7 illustrates the incidence of Grade ≥ 3 TRAEs associated with tislelizumab in patients with HCC, synthesizing data from six studies. The fixed-effect model yields a pooled logit estimate of -0.458 (95% CI: -0.631 to -0.264; SE = 0.094), which translates approximately to a 28.9% probability of experiencing TRAEs. This suggests that roughly one in three patients undergoing tislelizumab therapy may develop some form of adverse reaction attributable to the treatment. The Z-value of -4.78 (p < 0.000001) confirms the statistical significance of this finding. However, the analysis reveals substantial heterogeneity among studies, with a Q-value of 180.05, df = 5, and I² = 97.2%, indicating that nearly all the variability is due to real differences between studies rather than chance. The Tau² of 2.25 further confirms this inconsistency. As a result, the random-effects model provides a more conservative estimate, with a logit of -1.112, corresponding to a lower probability of adverse events (approximately 24.7%), though the 95% CI crosses zero, reducing its statistical strength. Qin et al. (2023) [23] and Li et al. (2024) [26] contributed substantially to the pooled estimate with significantly negative logits (-2.67 and -3.06), suggesting low TRAE rates. In contrast, Ren et al. (2023) [22] reported a positive logit (0.575), indicating a higher adverse event risk, which contributes to overall heterogeneity. Clinically, these findings underscore that tislelizumab is generally well tolerated, with an acceptable safety profile. However, the high heterogeneity suggests that TRAEs may vary significantly based on population characteristics or clinical context, warranting closer patient monitoring and tailored risk assessments in real-world settings.

Publication Bias

**Figure 8:**
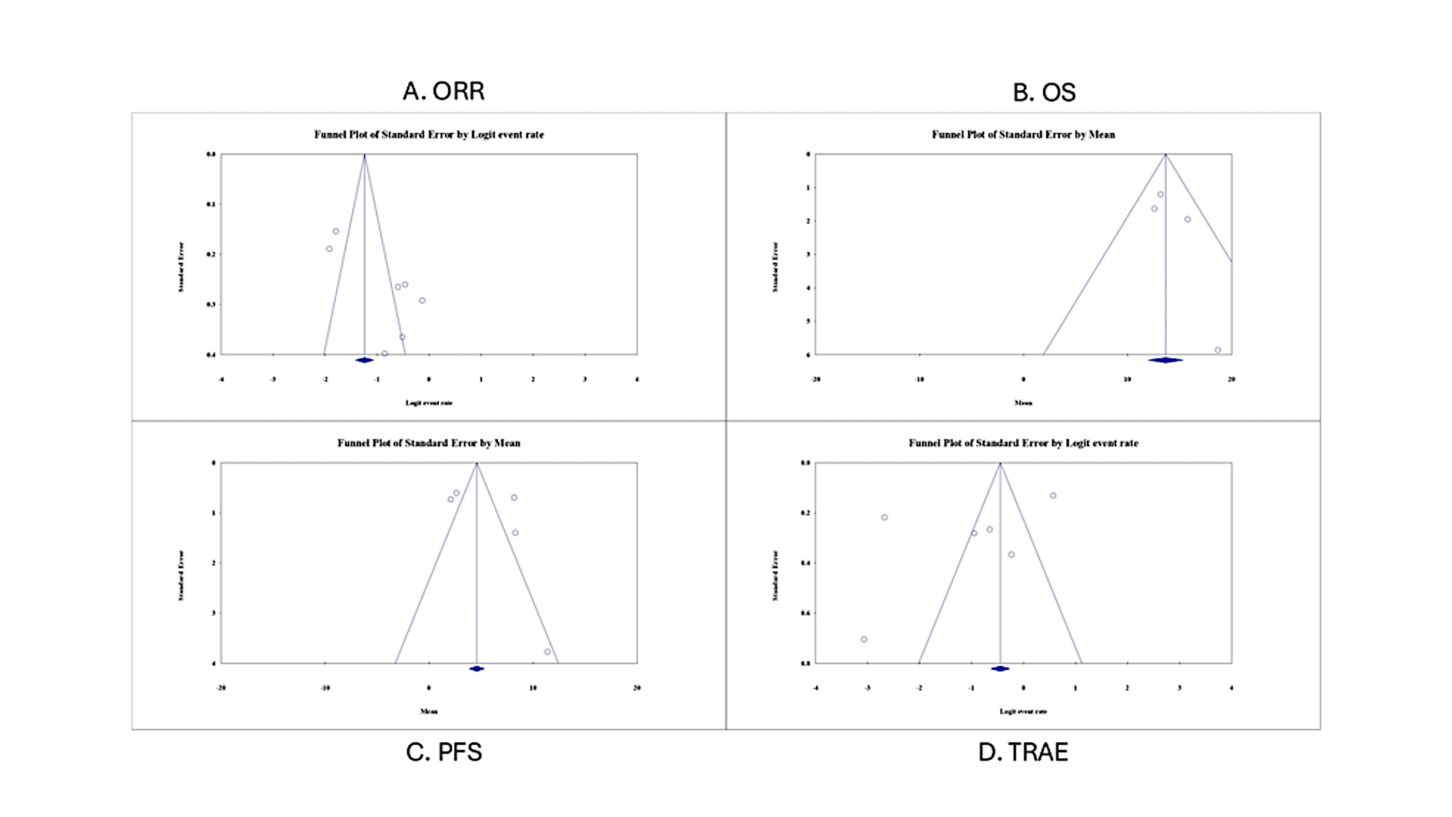
Funnel plots on publication bias for the included outcomes ORR: objective response rate; OS: overall survival; PFS: progression-free survival; TRAE: treatment-related adverse events

Figure 8 shows publication for the tislelizumab meta-analyses on OS, ORR, PFS, and TRAEs using a variety of methods, including Begg and Mazumdar's rank correlation test, Egger's test of the intercept, Duval and Tweedie's trim-and-fill method, and fail-safe N calculations. For OS, Begg's test yielded a non-significant Kendall's tau-b of 0.167 (p = 0.734), and Egger's test showed a non-significant intercept (p = 0.278), suggesting no strong evidence of publication bias. The trim-and-fill method identified one missing study, but the adjusted effect estimate was only slightly lower (13.55 vs. 13.66), indicating a minimal impact. In contrast, ORR analysis revealed signs of publication bias. Egger's test showed a statistically significant intercept (B0 = 7.61, p = 0.027), while Begg's test remained non-significant (tau-b = 0.190, p = 0.548). The trim-and-fill method imputed three missing studies, and the adjusted response rate dropped notably from 22.4% to 17.2%, indicating potential inflation of effect due to small-study bias. For PFS, Begg's (tau-b = 0.100, p = 0.807) and Egger's (B0 = 3.39, p = 0.477) tests were not significant, though the trim-and-fill method detected two missing studies, resulting in a minor decrease in point estimate (from 4.58 to 4.22 months), suggesting limited bias. Finally, for TRAEs, both Begg's (p = 0.707) and Egger's tests (p = 0.251) were non-significant, and the trim-and-fill method suggested no missing studies, affirming the robustness of this safety outcome. In summary, publication bias appears to be minimal for OS, PFS, and TRAE outcomes; however, bias is evident in the ORR estimates, where corrective methods, such as the trim-and-fill method, were applied to adjust the summary effect and ensure validity. These adjustments are crucial for guiding accurate clinical interpretations and evidence-based decision-making.

Discussion

This study comprehensively evaluates the clinical efficacy and safety of tislelizumab in treating HCC, providing insights into four key outcomes: ORR, OS, PFS, and TRAEs. For ORR, the fixed-effect model yielded a pooled logit effect size of -1.242, corresponding to an estimated response rate of 22.3%. However, high heterogeneity (I² = 89.93%) and a significant Egger's intercept (p = 0.027) indicate potential publication bias, which is supported by Duval and Tweedie's trim-and-fill analysis, estimating three missing studies. When these were imputed, the ORR dropped to 17.2%, suggesting inflation due to small-study effects. Clinically, while tislelizumab demonstrates a modest anti-tumor response, this may be somewhat overestimated, reinforcing the need for larger, high-quality trials to confirm efficacy. In contrast, OS outcomes were robust. The pooled survival was 13.66 months, with no evidence of heterogeneity (I² = 0%) or bias (Egger's p = 0.278; Begg's p = 0.734). The trim-and-fill adjusted values remained virtually unchanged. This consistency supports a genuine survival benefit, indicating that tislelizumab offers meaningful life extension in HCC patients. For PFS, the pooled estimate was 4.58 months, with high heterogeneity (I² = 93.15%). Although Egger's (p = 0.478) and Begg's tests (p = 0.807) did not indicate publication bias, the trim-and-fill method suggested two missing studies, slightly lowering the PFS to 4.22 months. This implies modest efficacy with potential variations influenced by population or trial factors.

Lastly, the TRAEs logit rate was -0.448, reflecting a 28.9% risk. Despite substantial heterogeneity (I² = 97.2%), no publication bias was found across methods, affirming the acceptable safety profile of tislelizumab. Notably, studies such as Ren et al. (2023) [24], which assessed combination therapies involving ociperlimab and BAT1706 alongside tislelizumab, may have contributed to elevated ORR and PFS estimates. These results likely reflect the additive or synergistic effects of multi-agent regimens and should not be interpreted as representative of tislelizumab monotherapy.

A comparison with the findings of Abushanab et al. [29], who assessed tislelizumab across various malignant solid tumors, offers valuable context. Their pooled analysis reported an HR of 0.71 (95% CI: 0.65-0.78) for OS, aligning with our results and reinforcing the survival benefit of tislelizumab. For PFS, they found an HR of 0.68 (95% CI: 0.54-0.84, p = 0.0005), indicating a similar reduction in disease progression. While their reported ORR was higher (2.59, 95% CI: 2.15-3.12), this likely reflects broader tumor types or combination regimens. Importantly, both studies demonstrated comparable treatment-related adverse event profiles, supporting the drug's favorable safety across indications. Together, these findings affirm tislelizumab's clinical utility, with differences in efficacy outcomes influenced by tumor biology and therapeutic context.

Lin et al.'s [30] analysis of neoadjuvant tislelizumab-based therapy in NSCLC demonstrated a higher pooled incidence of 89% for all-grade TRAEs and 17% for Grade ≥ 3 TRAEs, while our study reported a TRAE incidence of 19% for Grade ≥ 3. This demonstrates the safety profile of the drug in various cancer biology applications, showing similarity.

A comparison with other established immunotherapies, such as nivolumab and atezolizumab, provides important context for evaluating the clinical relevance of tislelizumab in HCC. Tislelizumab, a high-affinity anti-PD-1 antibody with a dissociation constant of 0.15 nmol/L and >90% receptor occupancy at 5 mg/kg, exhibits a half-life of 16.8 days, a clearance of 0.247 L/day, and robust pharmacological activity by activating tumor-infiltrating lymphocytes and inducing superior IFN-γ production compared to nivolumab and pembrolizumab, supporting its clinical evaluation [6,7]. The pooled ORR for tislelizumab was approximately 22.3%, adjusted to 17.2% after accounting for potential publication bias. This response is comparable to that reported in the CheckMate-040 [31] trial, where nivolumab, another PD-1 inhibitor, demonstrated an ORR of 15% in advanced HCC patients post-sorafenib failure [31]. Although slightly lower, the similarity in ORR between the two agents supports the consistent class effect of PD-1 blockade, indicating that tislelizumab provides competitive therapeutic potential as monotherapy.

In terms of OS, tislelizumab showed a pooled estimate of 13.66 months, a result that aligns with the IMbrave150 trial [32], where atezolizumab in combination with bevacizumab achieved a median OS of 13.2 months [32]. Despite the IMbrave150 regimen involving combination therapy and a broader patient base, the comparable survival outcome underscores the potential of tislelizumab to offer significant life extension in HCC patients as a monotherapy. Regarding PFS, tislelizumab yielded a pooled estimate of 4.58 months (random effects: 5.86 months), whereas atezolizumab plus bevacizumab achieved a median of 6.8 months. While this favors combination regimens, tislelizumab still offers meaningful disease control. TRAEs occurred in 28.9% of tislelizumab-treated patients, comparable to nivolumab (19%) and atezolizumab-based regimens (30%) [31,32], confirming a manageable safety profile. Collectively, these comparisons reinforce tislelizumab's viability as a monotherapy option, especially where combination therapies may not be appropriate due to cost, comorbidities, or tolerability concerns.

The study on tislelizumab for HCC faces several limitations. First, significant heterogeneity exists among study populations, with variations in patient demographics, tumor stages, and prior treatments, limiting the generalizability of the findings. Second, the inclusion of both RCTs and observational studies introduces methodological variability, which impacts the robustness of the pooled outcomes. Third, moderate to high risks of bias in areas such as confounding and outcome measurement reduce the reliability of the results, particularly in non-randomized studies. Fourth, small sample sizes in several included studies lower the statistical power and precision of the meta-analysis. Fifth, publication bias is evident in some outcomes, particularly the ORR and TRAEs, which could overestimate the drug's efficacy or safety. Sixth, a lack of long-term follow-up in most studies prevents an assessment of tislelizumab's sustained efficacy and safety. Lastly, variations in how key outcomes are defined and measured, such as tumor responses and adverse events, hinder direct comparisons and the drawing of robust conclusions. Addressing these limitations in future research is crucial to validate the findings and optimize the clinical use of tislelizumab.

Future studies on tislelizumab for HCC should address the limitations of current research to enhance its clinical applicability. First, larger, multicenter RCTs with diverse populations are needed to improve the generalizability of findings and reduce variability caused by demographic and clinical heterogeneity. Ensuring consistent patient inclusion criteria, such as tumor stage and prior treatments, can also enhance the comparability of results across studies. Second, standardized definitions and measurements for key outcomes, such as tumor responses, PFS, and TRAEs, should be adopted to ensure reliable meta-analytical conclusions. Long-term follow-up data are crucial to evaluate the durability of tislelizumab's therapeutic effects and safety profile. Third, advanced statistical techniques, such as subgroup analyses and propensity score matching, could help identify patient subgroups that may derive the most benefit, aiding personalized treatment approaches. Addressing publication bias through pre-registration of clinical trials and transparent reporting of all results, including non-significant findings, is essential. Finally, exploring tislelizumab's efficacy in combination with other therapies, such as targeted agents or radiotherapy, could unlock synergistic effects and broaden its therapeutic potential. Incorporating real-world evidence will further enhance the understanding of its effectiveness and safety in routine clinical practice.

## Conclusions

This meta-analysis provides a comprehensive evaluation of tislelizumab's clinical performance in the treatment of HCC across four critical endpoints: ORR, OS, PFS, and TRAEs. The pooled ORR using the fixed-effect model was 22.3% (logit = -1.242, SE = 0.0897), indicating a modest but clinically meaningful tumor response. However, evidence of publication bias was detected through Egger's test (p = 0.027) and confirmed by the trim-and-fill method, which adjusted the ORR downward to 17.2%, suggesting some inflation due to small-study effects. Despite this, the response rate remains within the range of similar PD-1 inhibitors like nivolumab, which has shown a 15% ORR in similar patient populations. The OS analysis yielded a robust pooled estimate of 13.66 months (95% CI: 11.97-15.35), with no heterogeneity (I² = 0%) or publication bias (Egger's p = 0.278), reinforcing the reliability of this outcome. This is on par with or even favorable to survival results from combination regimens such as atezolizumab plus bevacizumab in the IMbrave150 trial (13.2 months), supporting tislelizumab's clinical value even as monotherapy. For PFS, the fixed-effect estimate was 4.58 months (CI: 3.85-5.31), increasing to 5.86 months under the random-effects model. However, high heterogeneity (I² = 93.15%) and modest bias (due to two studies imputing values) suggest response variability across subpopulations. Meanwhile, the incidence of Grade ≥ 3 TRAEs was estimated at 28.9% (logit = -0.448), aligning closely with safety profiles of nivolumab and atezolizumab, and with no evidence of publication bias detected.

Clinically, these results confirm that tislelizumab is an effective and well-tolerated immunotherapy option for HCC, particularly valuable in patients who are unsuitable for combination therapies. Its performance in survival and tolerability outcomes demonstrates meaningful clinical benefit, while observed heterogeneity in response outcomes highlights the need for stratified treatment approaches. Future large-scale trials and real-world studies should further refine patient selection criteria to maximize the therapeutic impact of tislelizumab in the treatment of advanced HCC.

## Appendices

GRADE assessment

**Table 3:** GRADE assessment GRADE: Grading of Recommendations Assessment, Development, and Evaluation

Study	Design	Risk of Bias	Inconsistency	Indirectness	Imprecision	Publication Bias	Overall GRADE
Ren et al. 2022 [22]	Non-randomized phase 2	Moderate: open-label design with no comparator	Serious: potential variability across 8 countries	No: direct HCC population	Moderate: large N, but low female %	Possible: non-randomized	Low
Qin et al. 2023 [23]	Randomized phase 3	Low: high-quality RCT	No: consistent reporting	No: directly applicable	Low: large sample (n=674)	Unlikely: well-powered RCT	High
Ren et al. 2023 [24]	Randomized phase 2	Moderate: small sample, open-label	Serious: multiple arms with different drugs	Yes: combination therapy limits generalizability	Serious: n=94	Possible: limited geographic scope	Low to Moderate
Li Z et al. 2023 [25]	Phase 1b, single center	Serious: very small sample, no control	Serious: exploratory in early-stage HCC	Yes: population differs (early-stage, not advanced HCC)	Serious: very low N=20	Likely: early-phase, single-site	Very Low
Li J et al. 2024 [26]	Single-arm phase II	Moderate: no control group	Serious: unique combination therapy	Yes: indirect comparison due to added agents	Moderate: n=47	Possible: single-center design	Low
Pan et al. 2024 [27]	Single-arm phase 2	Moderate: lacks control	Serious: limited generalizability (macrovascular invasion)	Yes: special subset of HCC	Serious: n=30	Possible: small size	Very Low to Low
Xu et al. 2024 [28]	Single-arm phase 2	Moderate: open-label, no comparator	Moderate: homogeneous Chinese population	No: applicable population	Moderate: n=64	Possible: limited geography	Low to Moderate
